# Responses of Digestive, Antioxidant, Immunological and Metabolic Enzymes in the Intestines and Liver of Largemouth Bass (*Micropterus salmoides*) under the Biofloc Model

**DOI:** 10.3390/antiox13060736

**Published:** 2024-06-17

**Authors:** Yuqin Jin, Shunlong Meng, Huimin Xu, Chao Song, Limin Fan, Liping Qiu, Dandan Li

**Affiliations:** 1 Wuxi Fisheries College, Nanjing Agricultural University, Wuxi 214128, China; jinyq@stu.njau.edu.cn (Y.J.); songc@ffrc.cn (C.S.); fanlm@ffrc.cn (L.F.); 2 Freshwater Fisheries Research Center, Chinese Academy of Fishery Sciences, Risk Assessment Laboratory for Environmental Factors of Aquatic Product Quality and Safety of the Ministry of Agriculture, Key Open Laboratory of Inland Fishery Ecological Environment and Resources, Wuxi 214081, China; xuhuimin@ffrc.cn (H.X.); qiulp@ffrc.cn (L.Q.); lidandan@ffrc.cn (D.L.)

**Keywords:** biofloc, largemouth bass, digestive, antioxidant, immune, glucose metabolism

## Abstract

To investigate the activities of intestinal digestive enzymes, liver antioxidant enzymes, immunological enzymes, and glucometabolic enzymes in largemouth bass (*Micropterus salmoides*) under the biofloc model, an experiment was conducted in 300-liter glass tanks. The experiment comprised a control group, which was fed a basal diet, and a biofloc group, where glucose was added to maintain a C/N ratio of 15. Each group had three parallel setups, with a stocking density of 20 fish per tank. The experiment ran for 60 days, employing a zero-water exchange aquaculture model. The results showed that at the end of the culture period, there were no significant differences between the initial weight, final weight, WGR, SGR, and SR of the biofloc group and the control group of largemouth bass (*p* > 0.05), whereas the lower FCR and the higher PER in the biofloc group were significant (*p* < 0.05); intestinal α-amylase, trypsin, and lipase activities of largemouth bass in the biofloc group were significantly increased by 37.20%, 64.11%, and 51.69%, respectively, compared with the control group (*p* < 0.05); liver superoxide dismutase and catalase activities, and total antioxidant capacity of largemouth bass in the biofloc group were significantly increased by 49.26%, 46.87%, and 98.94% (*p* < 0.05), while the malondialdehyde content was significantly reduced by 19.91% (*p* < 0.05); liver lysozyme, alkaline phosphatase, and acid phosphatase activities of largemouth bass in the biofloc group were significantly increased by 62.66%, 41.22%, and 29.66%, respectively (*p* < 0.05); liver glucokinase, pyruvate kinase, glucose-6-phosphate kinase, pyruvate kinase, glucose-6-phosphatase, and glycogen synthase activities were significantly increased by 46.29%, 99.33%, 32.54%, and 26.89%, respectively (*p* < 0.05). The study showed that the biofloc model of culturing largemouth bass can not only enhance digestive enzyme activities, antioxidant capacity, and immune response but can also promote the process of glucose metabolism and reduce feeding costs. This study provides data support for healthy culturing of largemouth bass in future production, provides a theoretical reference for optimizing the biofloc technology culture model, and is crucial for promoting the healthy and green development of aquaculture.

## 1. Introduction

Largemouth bass (*Micropterus salmoides*), commonly known as California bass, is native to North America and is one of the world’s most productive freshwater carnivorous fish. In the 1970s, largemouth bass was introduced into Taiwan, China, and then into Guangdong through artificial breeding. Due to its advantages of fast growth, strong adaptability, good meat quality, and disease resistance, it has become one of the most important aquaculture species in China after half a century of development [1,2]. According to the 2023 China Fisheries Statistical Yearbook, in 2022, China’s freshwater perch aquaculture production reached 802,500 t, an increase of 14.30% from 2021. Although the products of perch aquaculture are beneficial to society, the development of intensive and semi-intensive aquaculture methods has caused problems such as deterioration of aquaculture water quality, frequent occurrence of diseases, and environmental degradation, which limit the sustainable development of perch aquaculture in China.

Biofloc technology (BFT) has been widely used as a feasible and environmentally friendly alternative to traditional aquaculture [3]. Avnimelech first systematically proposed the concept of bioflocs and pointed out that the addition of carbon sources to maintain a suitable C/N ratio in the aquaculture system was the core of the BFT aquaculture model [4]. The BFT aquaculture system contains a large number of bioflocs, which are floc-like substances that are aggregated by aerobic microorganisms through flocculation of bacteria, algae, feed residues, and some inorganic substances in the water column [5]. In the BFT culture system, only a small amount of water is exchanged due to evaporation and removal of excess bioflocs [3]; the microorganisms in the culture system reuse waste nitrogen [6] and the flocs can be ingested by farmed animals, hence it has the benefits of protecting the environment, regulating water quality, and promoting the recycling of materials. Currently, largemouth bass are mainly cultured in ponds, and the water quality is maintained through large water exchange and good economic benefits have been achieved; however, this method has the disadvantages of polluting the ecological environment and wasting water resources [7]. The biofloc model does not change the water during the aquaculture process (only replenishes the evaporated water), thus reducing the pollution of sewage discharge to the environment. It also reduces harmful factors in the external environment from being introduced into the aquaculture organisms. Additionally, the biofloc is used to maintain the stability of the aquaculture system’s water quality and to control ammonia nitrogen, while nitrite nitrogen is kept at a low level. Furthermore, it reduces the morbidity rate or eliminates morbidity so that the aquaculture organisms can grow healthily and rapidly.

The biofloc technology culture model is one of the new culture models for effectively solving current aquaculture problems that has been successfully applied to intensive fish culture, including carp (*Cyprinus carpio*), Nile tilapia (*Oreochromis nilotica*), silver carp (*Hypophthalmichthys molitrix*), and bighead carp (*Aristichtys nobilis*) [8]. Some studies have shown that the biofloc culture model can not only improve the efficiency of feed utilization but can also improve the condition of the liver of cultured organisms and improve digestion and immunity, thus promoting the overall healthy growth of cultured organisms [9]. Up to now, most researchers have conducted relevant studies on the application of biofloc technology, including investigating the effects of biofloc on digestive enzyme activity [10], antioxidant capacity [11], and non-specific immune function [12] of shrimp (*Penaeus orientalis*); monitoring digestive enzyme activity and immune response of golden crucian carp (*Carassius auratus*) in the biofloc system [13]; and investigating the effects of biofloc on the immune and antioxidant status of Nile tilapia [14,15]. However, there are fewer studies on culturing largemouth bass using the biofloc technology culture model, and a large part of the unknown status of digestive, antioxidant, immune, and metabolic capacities of largemouth bass in the biofloc culture model is unexplored and undescribed. Therefore, this study aims to investigate the effects of the biofloc culture system on intestinal digestive enzymes, liver antioxidant enzymes, immune enzymes, and glucose metabolism enzymes of largemouth bass to provide data support for the healthy culture of largemouth bass in future production and to provide a theoretical reference for the optimization of the biofloc technology culture model, which is crucial for promoting the healthy and green development of aquaculture and accelerating the transformation and upgrading of the culture model.

## 2. Materials and Methods

### 2.1. Experimental Design

This experiment was conducted at the Freshwater Fisheries Research Center of the Chinese Academy of Aquatic Sciences (CAAS). The experiment was conducted in 300-liter glass tanks. The experiment comprised a control group, which was fed a basal diet, and a biofloc group, where glucose was added to maintain a C/N ratio of 15. Each group had three parallel setups, with a stocking density of 20 fish per tank. The experiment was run for 60 days (August–October 2023), employing a zero-water exchange aquaculture model, during which time only the water lost due to sampling and natural evaporation was replenished [16]; however, when fish in the control group stopped feeding or were feeding minimally, 25% of the total volume of water was replaced. Prior to the experiment, a small amount of pond substrate was placed at the bottom of each tank in the biofloc group to provide indigenous microorganisms that helped to promote the formation of biofloc. Two aeration heads were added to each cylinder, placed on both sides of the cylinder, and connected to the SHZ-D (III) circulating water multi-purpose vacuum pump to increase oxygen and stir the water.

### 2.2. Feeding and Management of Experimental Fish

The largemouth bass used in the experiment was purchased from Jiangsu Zhongshui Dongze Agricultural Development Co., Ltd. (Wuxi, China). After a 14-day culture acclimatization period, healthy, uniformly sized largemouth bass (average weight 33.26 ± 1.18 g) were selected and each tank was stocked with 20 fish. The bass were fed commercial feed (46.00% crude protein, 6.00% crude fiber, 6.00% crude fat, 16.00% crude ash, 1.20% total phosphorus, 2.30% lysine, and 12.00% moisture) at 3% of their body weight twice a day (9:00 a.m. and 5:00 p.m.), and 6 fish were randomly weighed every 15 days to adjust the feed intake. During the experiment, the water temperature was kept at 24–30 °C, pH was 6.5–8.5, and dissolved oxygen was maintained at 7–9 mg/L. Biofloc volume (in mL) was determined using Imhoff cones.

### 2.3. Experimental Carbon Source and Addition Method

The glucose used in the test was dextrose monohydrate (purchased from Renhe Tang Pharmaceutical Co., Ltd., Linyi, China). The carbon content was 36.37%. The amount of glucose added was based on the nitrogen content of the feed in the biofloc group, and the C/N ratio was 15:1, according to Avnimelech [4]. Glucose was added as follows: 30 min after feeding every day, glucose was dissolved in a small amount of cultured water and then splashed evenly in the experimental tanks.

### 2.4. Sample Collection

Samples were taken 0, 15, 30, 45, and 60 days from the start of the culture experiment and largemouth bass were fasted for 24 h. Two fish were randomly selected from each tank and six fish were taken from the biofloc group and the control group. The fish were anesthetized using MS-222 fish anesthetics at a concentration of 70 mg/L. Then, all the fish from each tank were weighed and the final weights were used to calculate growth and feed utilization parameters. After the fish were fully anesthetized, they were dissected and their livers and intestines taken out, placed in 2 mL centrifugal tubes, and placed in a refrigerator at −80 °C for storage. The livers and intestines were chopped and weighed (accurate to 0.01 g), diluted 10× with precooled phosphate-buffered saline, and homogenized on ice, then centrifuged at 12,000× *g* for 10 min at 4 °C, after which each supernatant was aliquoted into a centrifuge tube and immediately stored at −80 °C for physiological parameter analysis.

### 2.5. Growth Performance and Survival

The parameters, including the weight gain rate (WGR, %), specific growth rate (SGR, % day^−1^), survival rate (SR, %), feed conversion ratio (FCR), and protein efficiency ratio (PER, %), were studied to determine fish growth performance:

Weight gain rate (WGR, %) = (final weight − initial weight) × 100/initial weight

Specific growth rate (SGR, % day^−1^) = (In final weight − In initial weight) × 100/days

Survival rate (SR, %) = final number of fish × 100/initial number of fish

Feed conversion ratio (FCR) = total diet fed/(final weight − initial weight)

Protein efficiency ratio (PER, %) = (final weight − initial weight) × 100/protein intake

### 2.6. Liver and Intestinal Biochemical Analyses

Intestinal α-amylase (α-AL), trypsin (TRP), lipase (LPS), liver superoxide dismutase (SOD), catalase (CAT), Lysozyme (LZM), alkaline phosphatase (AKP), acid phosphatase (ACP), glucokinase (GK), pyruvate kinase (PK), glucose-6-phosphatase (G6PC), and glycogen synthase (GCS) activities, liver malondialdehyde (MDA) content, and total antioxidant capacity (T-AOC) were detected using commercial assay kits (Shanghai Enzyme Link Biotechnology Co., Ltd. Shanghai, China). Soluble protein concentrations of all enzyme extracts were determined according to Bradford [17]. All enzyme assays were conducted in triplicate in a 96-well microplate and carried out at 25 °C using the MicroStation Identification System (Biolog Inc. Hayward, CA, USA).

### 2.7. Statistical Analysis

The obtained parameters were analyzed and organized using Excel 2021 and the results of all data analysis were expressed as mean ± standard deviation (Mean ± SD). Shapiro–Wilk and Levene’s tests were used to analyze the normality and variance homogeneity of the data, then the control and biofloc groups were compared using an independent samples *t*-test. Analysis was conducted using SPSS statistics 27.0 software (SPSS Inc., Chicago, IL, USA), and *p* < 0.05 was the accepted significance level.

## 3. Results

### 3.1. Growth Performance and Feed Utilization

Growth performance and feed utilization of largemouth bass reared in the control and biofloc groups are shown in Table 1. There were no significant differences between the initial weight, final weight, WGR, SGR, and SR of the biofloc group and the control group of largemouth bass (*p* > 0.05), whereas the FCR and PER were significantly affected by the culture system (*p* < 0.05). The FCR of the biofloc group was lower than that of the control group, and the PER of the biofloc group was higher than that of the control group.

### 3.2. Digestive Enzymatic Activities in the Intestines

**Figure 1:**
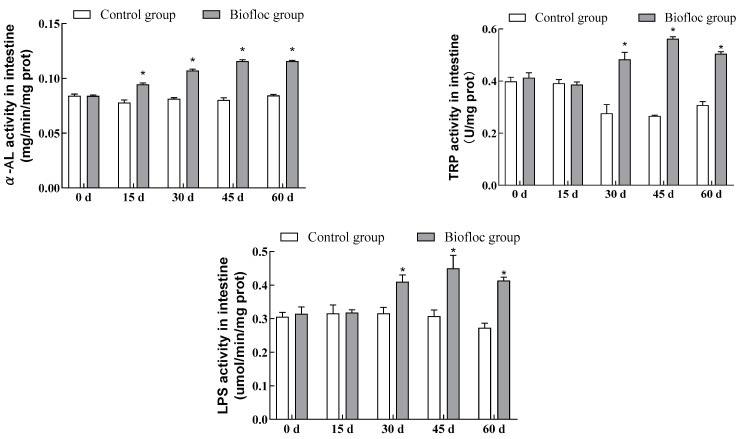
Digestive parameters (α-AL, TRP, LPS) in the intestines of largemouth bass in the control group and the biofloc group at the end of the 60-day experiment. Each value represents the mean ± SD. “*” means a significant difference from the control, with *p* < 0.05 being considered significant.

The commercial assay kits were utilized to detect the intestinal α-AL, TRP, and LPS activities of largemouth bass (Figure 1). From the results of the tests, activities of intestinal α-AL, TRP, and LPS in the fish in the biofloc group showed an increasing trend during the culture experiment. There was no significant difference between the intestinal α-AL activity in the biofloc group and in the control group of largemouth bass on day 0 of culturing (*p* > 0.05), whereas intestinal α-AL activity in the largemouth bass in the biofloc group was significantly higher than in the largemouth bass in the control group on the 15th, 30th, 45th, and 60th days of culturing (*p* < 0.05). There was no significant difference (*p* > 0.05) between intestinal TRP and LPS activities of largemouth bass in the biofloc group and the control group on days 0 and 15 of culturing, while on the 30th, 45th, and 60th days of culturing, intestinal TRP and LPS activities of largemouth bass in the biofloc group were significantly higher than those of largemouth bass in the control group (*p* < 0.05). At the end of the culture period, the intestinal α-AL, TRP, and LPS activities of largemouth bass in the biofloc group had increased by 37.20%, 64.11%, and 51.69%, respectively, relative to the control group.

### 3.3. Antioxidant Enzymatic Activities in the Liver

**Figure 2:**
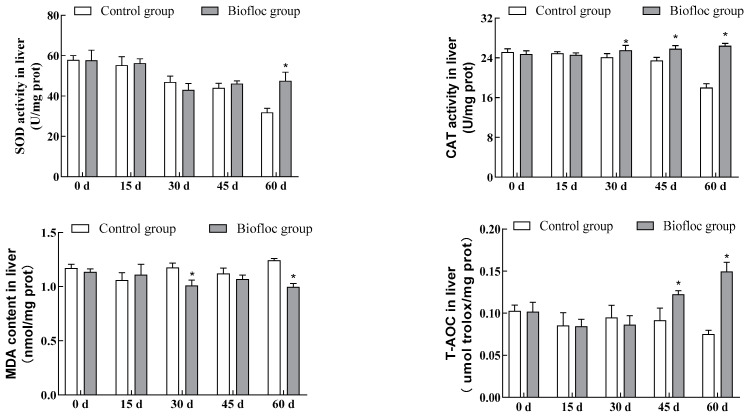
Antioxidant parameters (SOD, CAT, MDA, and T-AOC) in the liver of largemouth bass in the control group and the biofloc group at the end of the 60-day experiment. Each value represents mean ± SD. “*” means a significant difference from the control, with *p* < 0.05 being considered significant.

The commercial assay kits were used to detect SOD and CAT activities as well as MDA content and T-AOC in the livers of largemouth bass (Figure 2). The results showed that during the experimental period, SOD activity in the liver of largemouth bass in the biofloc group decreased and then increased, whereas the activity in the control group decreased consistently, and on the 60th day of cultivation, relative to the control group, SOD activity in the liver of largemouth bass in the biofloc group increased by 49.26% (*p* < 0.05). CAT activity in the liver of largemouth bass in the biofloc group showed a continuously increasing trend, whereas activity in the control group decreased continuously. CAT activity in the liver of largemouth bass in the biofloc group was significantly higher than in the liver of largemouth bass in the control group on the 30th, 45th, and 60th days of culturing (*p* < 0.05), and at the end of the culture period, CAT activity in the liver of largemouth bass in the biofloc group increased by 46.87% compared with that of largemouth bass in the control group (*p* < 0.05). The MDA content in largemouth bass liver in the biofloc group showed a decreasing trend, while that of the control group showed an increasing trend, and there was a significant difference between the biofloc group and the control group on the 30th and 60th days of culturing (*p* < 0.05); at the end of the culture period, the MDA content in largemouth bass liver in the biofloc group decreased by 19.91% compared with the control group. On days 0, 15, and 30 of culturing, there was no significant difference (*p* > 0.05) between largemouth bass liver T-AOC in the biofloc group and the control group, while at the end of the culture period, the largemouth bass liver T-AOC in the biofloc group was significantly higher than in the control group (*p* < 0.05). At the end of the culture period, largemouth bass liver T-AOC in the biofloc group increased by 98.94% compared with the control group.

### 3.4. Immunoenzymatic Activities in the Liver

**Figure 3:**
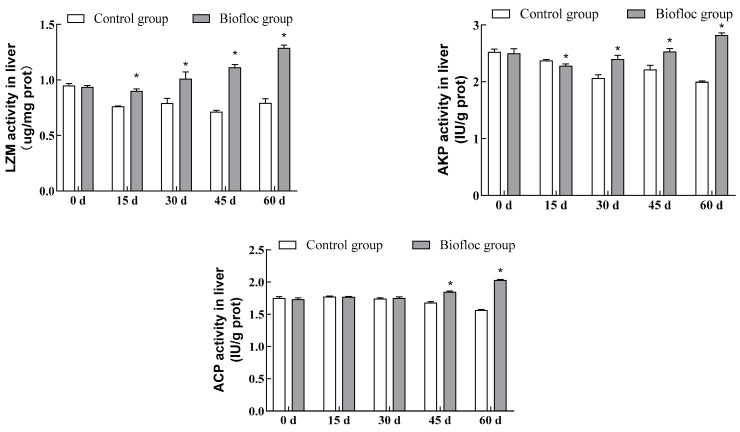
Immune-related parameters (LZM, AKP, ACP) in the liver of largemouth bass in the control group and the biofloc group at the end of the 60-day experiment. Each value represents mean ± SD. “*” means a significant difference from the control, with *p* < 0.05 being considered significant.

The commercial assay kits were used to detect LZM, AKP, and ACP activities in the liver of largemouth bass (Figure 3). It can be seen from the experimental results that, as the culture progressed, LZM activity in largemouth bass liver in the biofloc group showed a tendency to increase, while that in largemouth bass liver in the control group showed a tendency to decrease, except for day 0 of the experiment. At other stages, LZM activity in largemouth bass liver in the biofloc group was significantly higher than in the control group (*p* < 0.05), and at the end of the culture period, LZM activity in largemouth bass liver in the biofloc group increased by 62.66% compared to the control group; similarly, at other stages, except on day 0, there was a significant difference (*p* < 0.05) between the biofloc group and the control group in terms of largemouth bass liver AKP activity, and on the 60th day of culturing, the biofloc group’s largemouth bass liver AKP activity was increased by 41.22%; on days 0, 15, and 30 of culturing, there was no significant difference (*p* > 0.05) between the biofloc group and the control group in terms of largemouth bass liver ACP activity, while on the 45th and 60th days of culturing, liver ACP activity of the largemouth bass in the biofloc group was significantly higher than that of largemouth bass in the control group (*p* < 0.05). Liver ACP activity of largemouth bass in the biofloc group increased by 29.66% by the end of the culture period.

### 3.5. Metabolic Enzymatic Activities in the Liver

**Figure 4:**
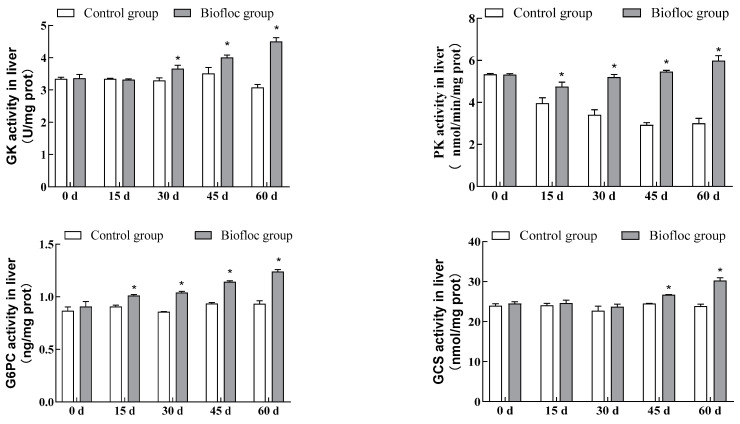
Metabolic parameters (GK, PK, G6PC, GCS) in the liver of largemouth bass in the control group and the biofloc group at the end of the 60-day experiment. Each value represents mean ± SD. “*” means a significant difference from the control, with *p* < 0.05 being considered significant.

The commercial assay kits were used to detect GK, PK, G6PC, and GCS activities in the liver of largemouth bass (Figure 4). From the test results, it can be seen that during the culture experiment, liver GK activity of largemouth bass in the biofloc group showed an increasing trend, and on the 30th, 45th, and 60th days of culturing, the liver GK activity of largemouth bass in the biofloc group was significantly higher than that of largemouth bass in the control group (*p* < 0.05). Liver PK activity of largemouth bass in the biofloc group showed a tendency to decrease and then increase, whereas that of the control group showed a tendency to decrease, except on day 0 of culturing. At other stages, the PK activity of largemouth bass liver in the biofloc group was significantly higher than in the control group (*p* < 0.05); similarly, except for day 0 of culturing, the G6PC activity of largemouth bass liver in the biofloc group was significantly higher than in the control group (*p* < 0.05); on days 0, 15, and 30 of culturing, there were no significant differences in the activity of largemouth bass liver GCS between the biofloc and the control groups (*p*> 0.05), while on the 45th and 60 days of culturing, the liver GCS activity of largemouth bass in the biofloc group was significantly higher than that of largemouth bass in the control group (*p* < 0.05). At the end of the culture period, the liver GK, PK, G6PC, and GCS activities of largemouth bass in the biofloc group increased by 46.29%, 99.33%, 32.54%, and 26.89%, respectively, relative to the control group.

## 4. Discussion

Substantial experimentations have shown that the biofloc system is able to not only enhance feed efficiency and provide high-quality protein sources but is also rich in growth promoters and bioactive compounds [18,19,20]. In our study, there was no significant difference between the growth of fish in the biofloc group and the control group. Interestingly, the FCR of the biofloc group was lower than that of the control group, and the PER of the biofloc group was higher than that of the control group, consistent with the results of Liu et al. [21] and Yu et al. [22]. The lower FCR and the higher PER values in the biofloc group might indicate biofloc consumption by the fish. This suggests that the biofloc system could play a certain role in feed substitution and could be applied in practical production to reduce feeding costs. The positive effects of the biofloc system on growth performance and feed utilization by fish might be explained by different factors, for example, biofloc provided good and stable water quality (in our previous study, the concentrations of ammonia and nitrite nitrogen in the biofloc group were significantly lower than in the control group [23]), biofloc could be continuously harvested by the fish as a good quality food source, and biofloc environment and consumption improved fish resistance against stress [24,25].

Digestive enzyme activity is one of the important bases for measuring the ability of fish to utilize bait and absorb nutrients. It can reflect the adaptive ability of fish to different environments, and at the same time, directly or indirectly determine the growth performance of fish and affect the growth of fish in later stages of life [26]. Changes in digestive enzyme activities in fish can reflect their digestive physiological characteristics to a certain extent. Biofloc can enhance the digestive ability of aquatic animals and, to a certain extent, can increase their digestive enzyme activities [21]. Amylase in the digestive tract mainly digests carbohydrates and sugars in food; amylase is a hydrolytic enzyme that has the ability to catalyze the hydrolysis of starch and glycogen to produce glucose, maltose, and a range of restricted dextrins containing branched chains of α-1,6-glycosidic bonds. The results of the present study showed that biofloc significantly increased intestinal α-amylase activity of largemouth bass. Xu et al. [10] found that amylase activity in the biofloc group was higher than in the control group, and Long et al. [27] demonstrated that biofloc increased intestinal amylase activity in tilapia (*Oreochromis* spp.). These are consistent with the results of the present study. Trypsin is an important protein hydrolase that plays a key role in protein digestion, hydrolysis, and utilization and is one of the important indicators for evaluating the growth and nutritional status of aquatic animals. In the present study, biofloc increased the intestinal trypsin activity of largemouth bass by 64.11% compared to the control group, which agreed with findings in Crayfish (*Procambarus clarkii*) [28], Rhododendron marsh shrimp (*Macrobrachium rosenbergii*) [29], and blunt snout bream (*Megalobrama amblycephala*) [30]. Lipase is mainly secreted by the hepatopancreas of fish and is capable of severing ester bonds. It can hydrolyze glycerol esters, phospholipids, and wax esters, which also occupy a very important position in the digestive physiology of fish. In the present study, lipase activity in the biofloc group was higher than in the control group, showing that biofloc can significantly increase lipase activity in the intestines of largemouth bass. Similarly, Yu et al. [13] showed that biofloc significantly increased the activity of intestinal lipase in golden crucian carp. However, in the study by Sun et al. [30] on the effect of biofloc on the digestive enzyme activity of blunt snout bream, there was no significant difference between intestinal lipase activity in the experimental group and the control group. This may be because the carbon sources added to the cultured biofloc were different, resulting in different compositions of flocs whose roles were also somewhat different.

It has been observed that largemouth bass ingest biofloc, and it has been shown that biofloc contain exogenous digestive enzymes such as amylase, lipase, and trypsin [31], hence it is hypothesized that a certain amount of biofloc ingested by the largemouth bass increases the number of digestive enzymes in the intestine, thus improving digestion and metabolism levels. In addition, it is also possible that the high production of extracellular enzymes in biofloc stimulates the animals to secrete more endogenous digestive enzymes [13]. Therefore, BFT can increase intestinal digestive enzyme activity in largemouth bass. On the one hand, biofloc interferes with the structure of the intestinal flora of largemouth bass, altering microbial sites that have been balanced, produced, or induced by the production of digestive enzymes [32]. On the other hand, BFT contains unicellular algae, plankton, bacteria, and particulate organisms; it also has a complex internal microbial community structure with a wide range of beneficial microorganisms [10].

The generation of reactive oxygen species (ROS) in fish is mainly due to the organism being subjected to environmental stress or bacterial or viral attacks, and the high accumulation of ROS alters the structure and function of the cells in the organism, which in turn causes lipid peroxidation, leading to dysfunctions in tissues and organs [33]. Excessive ROS can cause oxidative stress in the organism, and antioxidant reactions are a protective mechanism used by the organism when excess ROS accumulates, as they can effectively scavenge the overproduced reactive oxygen species in the organism. The antioxidant responses in fish, including both enzyme-participating and non-enzymatically-participating antioxidant responses, are essential to the health of the fish [34]. Total antioxidant capacity (T-AOC) is the total antioxidant level composed of various antioxidant enzymes and antioxidant substances, etc. It represents the level of scavenging for free radicals by the organism and is used as a measure of the antioxidant capacity of fish. In this study, the liver T-AOC of largemouth bass in the biofloc group showed higher levels at the end of the culture period, suggesting that biofloc can promote the scavenging of free radicals and enhance the antioxidant response of the fish. Yu et al. [13] assessed the antioxidant capacity of golden crucian carp using biofloc, and their study showed that T-AOC was significantly enhanced in the gills, liver, and intestines of the fish. This corroborated that biofloc improved the antioxidant capacity of the fish. Superoxide dismutase (SOD) is an important antioxidant enzyme that specifically scavenges reactive oxygen radicals in the organism and can convert superoxide anion into hydrogen peroxide to maintain the balance of ROS in the organism, playing an important role in preventing organismal damage. Catalase (CAT) is produced by peroxisomes and mitochondria and acts on metabolically produced hydrogen peroxide and converts it into water and oxygen, thus it is a redox enzyme that has an important role in the antioxidant defense mechanism in fish [35]. Lower levels of SOD and CAT activities indicate that a large number of reactive oxygen radicals are accumulating in the cells of the organism, which may lead to cellular damage, Shourbela et al. [36] cultivated biofloc using molasses as an externally added carbon source and applied it to a culture of Nile tilapia. They observed an increase in the activities of SOD and CAT in the fish and concluded that biofloc can reduce oxidative stress in the fish and maintain the fish’s health condition. Nageswari et al. [37] also reported a significant increase in SOD and CAT activities in Suchi catfish (*Pangasianodon hypophthalmus*) cultured using the biofloc model, indicating that resistance to oxidative stress was enhanced in the biofloc model, which acted as an effective antioxidant for the fish. Similarly, the results of the present study showed that biofloc significantly increased fish liver SOD and CAT activities. Although there was a decrease in SOD activity in the biofloc group during the pre-culture period, this might be due to the fact that the biofloc system was not yet stabilized and was in the stage of forming a large number of flocs, and the presence of a large number of biologically active compounds might have reduced the production of SOD, which is in line with the results obtained by Haridas et al. [38], who reported that hepatic SOD activity of tilapia was reduced in the biofloc model. Malondialdehyde (MDA) is the end product of lipid peroxidation in the organism. It is the result of an imbalance in the antioxidant system of the organism and has toxic effects on cells. The content of malondialdehyde can reflect the degree of cell damage, and it is used as an important indicator of oxidative stress in fish. Liu et al. [39] reported a significant decrease in MDA content in Nile tilapia under the biofloc model, which is the same as the results of the present study. This indicates that the fish have better lipid peroxidation defense and regulatory mechanisms under the biofloc model.

In the present study, SOD and CAT activities were increased, T-AOC was enhanced, and MDA content was reduced in the biofloc group compared to the control group, suggesting that biofloc plays a role in the enhancement of antioxidant capacity and maintenance of organismal health of largemouth bass. It has been reported that the microorganisms in the biofloc stimulated the antioxidant reaction, and the presence of certain bioactive substances, such as carobins, polysaccharides, chlorophylls, polyphenols, and vitamins, enhanced the oxidative stress response and antioxidant capacity of the fish [40,41].

Fish occupies a lower position among the vertebrates, and its immune system can be divided into specific immunity and non-specific immunity. Non-specific immunity is also called innate immunity; the innate immune system of fish includes anti-bacterial, anti-virus, and anti-parasite responses, of which the anti-bacterial response is the predominant response and is one of the most important differences between fish and other vertebrates [42]. The immune organs of fish include the liver, thymus, and head kidneys. Of these, the liver is an important place for metabolic enzymes that can rapidly transform the toxic substances produced during metabolic processes in the organism, reflecting the physiological and pathological conditions of the fish. Therefore, the liver has an important influence on fish health. Lysozyme (LZM) is an enzyme produced by leukocytes and plays an important role in the immune response of fish. It is the first line of defense against bacteria, viruses, and parasites, and not only stimulates the immune system of the fish to improve resistance and immunity but also inhibits the growth of gram-positive bacteria in the fish body, thus reducing the infection of fish by pathogenic bacteria [43]. Acid phosphatase (ACP) is an important antimicrobial molecule in the body and, along with LZM, is an important basis for evaluating the ability of the body to phagocytose pathogenic bacteria. Alkaline phosphatase (AKP) is involved in the transfer and metabolism of phosphate groups in the organism and enhances the ability of blood cells in the organism to recognize foreign substances. It is an important hydrolytic enzyme involved in the immune response in fish and plays an important role in maintaining the health of the fish [44]. LZM, ACP, and AKP play an important role in the innate immunity of the fish. In the present study, the liver LZM, ACP, and AKP activities of largemouth bass in the biofloc group were significantly higher than those of largemouth in the control group, indicating that biofloc has a positive effect on enhancing the immunity of the fish. Mansour and Esteban [14] reported a significant increase in the LZM activity of Nile tilapia in the biofloc model. Ahmad et al. [45] showed a significant increase in LZM and ACP activities in Roho labeo (*Labeo rohita*) in the biofloc system, suggesting that biofloc can enhance the non-specific immunity of cultured fish. Sun et al. [30] also reported that liver LZM and AKP activities of blunt snout bream in the biofloc group were significantly higher than in the control group, indicating that the biofloc culture technology has a certain effect on the immune activity of blunt snout bream. These findings are consistent with the results of this experiment. Many studies have shown that biofloc can enhance the LZM, ACP, and AKP activities of fish because of the presence of a large number of bioactive substances in biofloc, such as carotenoids, vitamins, phytosterols, and taurine, and the presence of probiotic microorganisms in the biofloc, which can enhance the immune response of the fish and provide protection for the organism [21,46,47]. On the other hand, fish change the structure of the digestive tract’s microflora by ingesting a certain amount of biofloc, which improves resistance to environmental stress and enhances fish immunity [48]. In addition, the biofloc culture system provides fish with a source of essential amino acids required by the organism, which, likewise, contributes to the enhancement of fish immunity in the biofloc culture system [49].

The main pathways of glucose metabolism in fish are glycolysis and gluconeogenesis. Glycolysis is the only way of breaking down glucose in fish. Glucokinase (GK) and pyruvate kinase (PK) are both key enzymes in the glycolytic pathway [50]. The increase in their activity indicates the increase in the body’s glycolytic activity, which, in turn, replenishes the body with the required energy. In fish, most of the glycolytic enzymes, including GK and PK, are involved in the glycolytic process in the liver. GK catalyzes the first step of glucose utilization by the organism, whereas PK is one of the rate-limiting enzymes in the glycolytic process. Ingestion of saccharides markedly promotes GK and PK activities [51]. Che et al. [52] reported that the ingestion of pea starch enhanced the hepatic GK activity of the largemouth bass, suggesting that the ingestion of pea starch could promote glucose utilization by largemouth bass. Cowey et al. [53] found that an increase in carbohydrate content in the diet increased the hepatic PK activity of rainbow trout (*Oncorhynchus mykiss*); Enes et al. [54] similarly found that the hepatic PK activity of the European seabass (*Dicentrarchus labrax*) increased significantly when the starch content in the diet was increased. Under the conditions of this experiment, liver GK and PK activities in largemouth bass in the biofloc group were significantly higher than in the control group, suggesting that the biofloc system can increase the utilization of glucose by largemouth bass, probably due to the fact that glucose was added to ensure the operation of the biofloc system in the present experiment, which indirectly resulted in a small amount of glucose ingestion by largemouth bass. Glycolysis is the conversion of non-glycans into glucose or glycogen and is not a simple inverse glycolytic reaction. Glucose-6-phosphatase (G6PC) catalyzes the synthesis of glucose directly and plays a catalytic role in the synthesis of glucose from hepatic glycogen, whereas glycogen synthase (GCS) is the rate-limiting enzyme in the process of glycogen synthesis, both of which play important roles in the process of gluconeogenesis. Changes in dietary carbohydrate content can affect changes in gluconeogenesis, especially in the activities of key gluconeogenic enzymes in the liver [55]. Lin et al. [56] reported that higher levels of dietary starch levels enhanced the activities of gluconeogenic enzymes, including G6PC in the liver of largemouth bass. In the present study, liver G6PC and GCS activities of largemouth bass in the biofloc group were significantly higher than those of largemouth bass in the control group, which indicated that the biofloc culture system had a facilitating effect on the glycolysis process in largemouth bass. It was speculated that the reason might be related to the addition of glucose to maintain the operation of the biofloc system, which resulted in the indirect intake of a small amount of glucose by the largemouth bass. It can be seen that biofloc can promote the glucose metabolism process in largemouth bass, which may be due to the following reasons: the addition of a carbon source, thus the increase in glucose metabolism-related enzyme activities after fish consumption or the presence of beneficial microorganisms and bioactive substances in the biofloc, which promotes the glucose metabolism process in fish.

In mammals, the increase in glycolytic enzyme activity is an adaptive response to elevated blood glucose, whereas the increase in gluconeogenic enzyme activity helps to maintain blood glucose levels [57]. In the present study, the increase in G6PC and GCS activities was lower than the increase in GK and PK activities, suggesting that the glycolytic process in largemouth bass is affected more by the carbohydrate intake than the gluconeogenic process. In addition, it is worth noting that some studies reported that high starch content in the feed led to liver damage and problems with gluconeogenesis in largemouth bass [58,59], thus the selection of carbon sources and the control of appropriate carbon-to-nitrogen ratios need to be considered when culturing largemouth bass using biofloc. Since there are few studies on the effects of biofloc on glucose metabolism in fish, the mechanism through which biofloc affects glucose metabolism in largemouth bass needs to be investigated further.

## 5. Conclusions

In conclusion, our results suggest that BFT has positive effects on digestive enzymes (α-AL, TRP, and LPS activities), antioxidant responses (SOD, CAT, MDA, and T-AOC), immunological enzymes (LZM, AKP, and ACP activities), and glucometabolic enzymes (GK, PK, G6PC, and GCS activities) in largemouth bass. Additionally, on the premise that growth differences were not significant, the lower FCR and the higher PER in the biofloc group showed that the biofloc model of culturing largemouth bass could not only enhance digestive enzyme activities, antioxidant capacity, and immune response, but also promote the process of glucose metabolism, and can reduce feeding costs. This study provides data support for the healthy culturing of largemouth bass in future production, provides a theoretical reference for optimizing the biofloc culture model technology, and is crucial for promoting the healthy and green development of aquaculture.

## Figures and Tables

**Table 1:** Growth performance and feed utilization of largemouth bass reared in the control and biofloc groups.

Parameters	Control Group	Biofloc Group
IW (g)	33.32 ± 1.30	33.18 ± 1.07
FW (g)	99.06 ± 5.39	97.15 ± 6.17
WGR (%)	196.46 ± 3.71	193.47 ± 6.65
SGR (% day^−1^)	1.81 ± 0.02	1.79 ± 0.04
SR (%)	93.33 ± 2.89	95.00 ± 0.00
FCR	1.48 ± 0.03 ^a^	1.14 ± 0.05 ^b^
PER (%)	1.47 ± 0.03 ^b^	1.90 ± 0.08 ^a^

Note: Data are mean ± SD. Numbers with different superscripted letters within the same row represent a significant difference (*p* < 0.05). Abbreviations: IW, initial weight; FW, final weight.

## Data Availability

All datasets generated for this study are available.
